# Frequent pattern mining in multidimensional organizational networks

**DOI:** 10.1038/s41598-019-39705-1

**Published:** 2019-03-01

**Authors:** László Gadár, János Abonyi

**Affiliations:** 1 Innopod Solutions Kft, Budapest, Hungary; 2 MTA-PE Budapest Ranking Research Group (BRRG), University of Pannonia, Veszprém, Hungary; 3 MTA-PE Complex Systems Monitoring Research Group, University of Pannonia, Veszprém, Hungary

## Abstract

Network analysis can be applied to understand organizations based on patterns of communication, knowledge flows, trust, and the proximity of employees. A multidimensional organizational network was designed, and association rule mining of the edge labels applied to reveal how relationships, motivations, and perceptions determine each other in different scopes of activities and types of organizations. Frequent itemset-based similarity analysis of the nodes provides the opportunity to characterize typical roles in organizations and clusters of co-workers. A survey was designed to define 15 layers of the organizational network and demonstrate the applicability of the method in three companies. The novelty of our approach resides in the evaluation of people in organizations as frequent multidimensional patterns of multilayer networks. The results illustrate that the overlapping edges of the proposed multilayer network can be used to highlight the motivation and managerial capabilities of the leaders and to find similarly perceived key persons.

## Introduction

In the early 1980’s Tichy suggested that organizational research should incorporate a network perspective^1,2^. In the 1990’s six themes (turnover and absenteeism, power, work attitudes, job design, leadership, motivation) dominated the research of micro-organizational behavior^3^. Researchers have highlighted that centrality in advice networks may differ from that of friendship networks. Advice network centrality is a better indicator of the “real” hierarchy^4^, because individuals may seek out advice from others who they would not consider leaders, and may perceive leaders whom they would not necessarily consider going to for advice^5^. Social influence derived from friendship networks has stronger effects on job-satisfaction^6^ because social network relationships are likely to affect the performance and receipt of organizational citizenship behavior (OCBs), which includes attitudes like job satisfaction. The spread of OCBs in organizations may be facilitated or hindered by social relationships^7^.

Social Network Analysis (SNA) is widely used to support these studies. As the attitude of the members of social networks attitudes might influence each other, predicting their behavior requires an advanced model of the connections^8^. The analysis of network reciprocity can also provide useful information about cooperation-promoting mechanisms^9^. The modeling forming connections is crucially important when we would like to understand how social networks evolve^10^. Recently it has been proven that combining the methods in game theory, agent-based modeling, machine learning, and computational sociology is a useful approach to understand the mechanisms of network formation^11^.

In organizational networks, the nodes are co-workers and ties are defined based on the researchers are focusing on. Edges can be defined based on communication^12^, advice^13^, friendship^14^ relationships, or all of these^15^. For getting a better understanding of what factors affect the formation of communication networks, connection types defined by the theory of structuration^16^ were shown to be useful^17^.

The importance of the multilayer nature of intra-organizational networks was realized more than thirty years ago^18,19^. The informal structure of an organization is complex and multilayered as people are involved in multiple, dynamic and overlapping webs of relationships^4^. In the early studies Multi-Theoretical Multi-Level models^20,21^ and multilayer networks^22–24^ were used to provide a deeper insight into organizations. The theory of multilayer networks^25,26^ is a rapidly growing field in network science. Nowadays multilayer networks are widely used in SNA^27–31^. Multiplex networks are a special case of multilayer networks where nodes are the same everywhere, and different edges lie on different layers^32^. In these models, the nodes can be characterized by their activities on different layers, which provides a better understanding of their roles^33–35^.

As can be seen, organizational networks have been considered to be multilayer networks since the early 1990s, but no method could handle the multidimensional aspect of the problem. Finding informative correlations between layers of multilayer networks is still considered as one of the primary goals of network science^26^.

The research aims to develop a methodology for the complex assessment of organizations that handles the multidimensional nature of the relationships.

The contributions of this work are the following:

- Based on our organizational development experience, requirements of our business partners, and the literature of organizational network analysis we defined a multilayer organization network with 15 layers representing interaction-, rating-/perception-, and friendship-type connections.
- The key idea behind the development of this model is that the connection types of the proposed multidimensional organizational network can be analyzed by association rule mining algorithm to reveal how relationships, motivations, and perceptions as layers of an organization influence each other in different scopes of activities.
- The novelty of our approach resides in the evaluation of people in organizations as frequent multidimensional patterns of incoming/outgoing multidimensional edges.
- We demonstrate that the overlapping edges of the proposed multilayer network can be used to highlight the motivation and managerial capabilities of the leaders and to find similarly perceived key persons.

The paper is organized as follows. In the first part of the Methods section, the multidimensional organizational network model is introduced. The second part of this section presents how frequent pattern mining can be used to extract information from multidimensional networks. It is believed that the proposed approach can be widely applied to find significant correlations between layers of multilayer networks. The Results and Discussion section demonstrates how the proposed approach can be used in the development of three organizations.

## Methods

### Multidimensional representation of organizational networks

**Figure 1:**
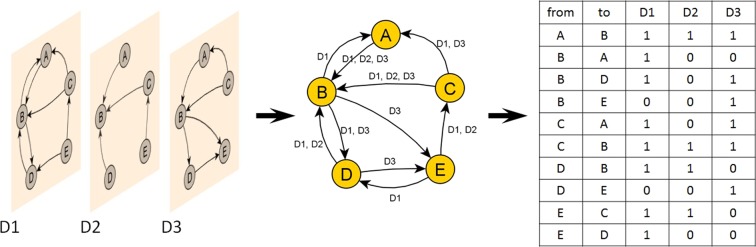
Representations of a multidimensional network.

In the proposed multidimensional organizational network the nodes represent the employees and labeled edges reflect how the members of the organization communicate, work together, rate and motivate each other, and their personal relationships. Labeled and directed connections define multiple edges form a multidimensional network ${\mathscr{G}}=(V,E,D)$, where *V* represents the node set, *D* the set of edge labels defines the dimensions of edges, and *E* denotes the edge set, $E=\{(u,v,d);u,v\in V,d\in D\}$ as can be seen in Fig. 1. As each label can be mapped into an independent network, the model can be interpreted as a multilayer network. A multilayer network is a pair ${\mathcal M} =({\mathscr{G}},{\mathscr{C}})$, where ${\mathscr{G}}=\{{G}_{\alpha };\alpha \in \{1,\ldots ,M\}\}$ is a family of graphs *G*_*α*_ = (*X*_*α*_, *E*_*α*_) (called layers of ${\mathcal M}$) and ${\mathscr{C}}=\{{E}_{\alpha \beta }\subseteq {X}_{\alpha }\times {X}_{\beta };\alpha ,\beta \in \{1,\ldots ,M\},\alpha \ne \beta \}$ is the edge set between nodes of different layers *G*_*α*_ and *G*_*β*_ with *α* ≠ *β*^26^. *E*_*α*_ are called intralayers and *E*_*αβ*_(*α* ≠ *β*) are referred to as interlayer-connections.

The studied intra-organizational networks can be considered to be directed multiplex networks which are a special type of multilayer networks. Multiplex networks consist of a fixed set of nodes connected by different types of links. In our case the *G* = (*V*, *E*, *D*) multidimensional network is associated with a multiplex network with layers $\{{G}_{1},\ldots ,{G}_{|D|}\}$ where *α* ∈ *D*, *G*_*α*_ = (*X*_*α*_, *E*_*α*_), *X*_*α*_ = *V*, *E*_*α*_ = {(*u*, *v*) ∈ *V* × *V*;(*u*, *v*, *d*) ∈ *E* and*d* = *α*}.

Based on our organizational development experience, requirements of our business partners, and the literature of organizational network analysis connection-/interaction-, rating-/perception-, and friendship-type layers were defined in our model:

1. Connection-type layers
  L1: get advice from
  L2: get priorities from
  L3: get feedback from
  L4: communication with
  L5: working together with
2. Rating-type layers
  L6: he/she helps to find information
  L7: he/she provides the best working relationship
  L8: he/she has great professional knowledge
  L9: he/she motivates me
  L10: he/she is capable of solving complex tasks
  L11: he/she is capable of managing colleagues
  L12: he/she is a key person in the organization
3. Friendship-network layers

L13: he/she gets along easily with me

L14: I would like to have dinner with him/her

L15: I would like to work together with him/her as a part of a problem-solving team

An online survey was designed to identify the connections. In the survey, there were as many questions as layers. Respondents were asked to mark the names of co-workers that fit the question and were not restricted to a fixed number of answers to minimize measurement error^36^.

The combination of layers is believed to capture the essence of an organization, making it possible to extract information about working connections, trust, employee’s perceptions of each other, and leadership.

### Frequent pattern mining of edge labels in multidimensional networks

Discovering statistically significant correlations between layers of multilayer networks is one of the major goals of network science over the next years^26^. A recently developed edge-overlap measure evaluates the conditional probability of finding a directed link on a layer given the presence of a directed link between the same nodes on another layer^34,37^ which can handle with pairs of dimension. The method is feasible for examining the overlap of a small number of dimensions. As the coexistence of links with different labels between any nodes *i* and *j* forms frequent patterns of any number of dimensions, it was found that frequent pattern mining provides a new opportunity to describe correlations between layers.

Frequent itemset mining was initially developed for market basket analysis, and it is used nowadays for almost any task that requires the discovery of regularities between (nominal) variables^38^. This concept has been extended to frequent graph-based substructure pattern mining^39^.

Our work differs from methods developed for frequent subgraph mining in unilayered (labeled) networks^40^. Labeling network motifs in protein-protein interaction (PPI) networks^41^ and text networks^42^ is also a similar problem. While in these tasks the labels are attached to the nodes, in our case the problem requires the identification and characterization of the frequent multidimensional edges.

As this is the first attempt to introduce frequent itemset mining into the analysis of multidimensional networks, the technique is summarized in Table 1. The dimensions *D* = {*d*_1_, *d*_2_, …, *d*_*M*_} of the network are considered to be a set of items *I* = {*I*_1_, *I*_2_, …, *I*_*M*_} (in market basket analysis, *I*_*i*_ represents a given product). The set of transactions of the items *T* = {*t*_1_, *t*_2_, …, *t*_*m*_} are defined as a set such that ${t}_{i}\subseteq I$ is identical to a given edge ${E}_{i}=\{({u}_{i},{v}_{j},d);u,v\in V,d\in D\}$ in a multigraph between nodes *u*_*i*_ and *v*_*j*_. Our aim is to identify frequently occurring subsets of edge dimensions and mine valuable information concerning multidimensional networks based on the analysis of these itemsets. The occurrence of an itemset *C* is measured as number of transactions (multidimensional edges) that the itemset contains. When this frequency is divided by the size of the transaction set $|{\mathscr{D}}|$ which is identical to the number of edges |*E*|, the calculated support of *s*_*T*_(*C*) represents the probability of multidimensional edge *C*. The $C\subseteq T$ is referred to as frequent when *s*_*T*_(*C*) ≥ *s*_*min*_ exceeds a user-specified minimum *s*_*min*_. The goal of frequent itemset mining is to find all frequent itemsets *C* ⊆ *I* in database ${\mathscr{D}}$^38^.

**Table 1:** Corresponding nomenclature of frequent itemset mining and multidimensional networks.

	Frequent itemset mining	Multidimensional network
Item base	*I* = {*I*_1_, *I*_2_, …, *I*_*M*_} *I*_*i*_, for example, represents a product	*D* = {*d*_1_, *d*_2_, …, *d*_*M*_} *d*_*i*_ is a dimension
Transaction	*T* = {*t*_1_, *t*_2_, …, *t*_*m*_} is a set of items *T* ⊆ *I*	*E*_*k*_ = {(*u*, *v*, *d*);*u*, *v* ∈ *V*, *d* ∈ *D*} is a multidimensional edge, which is a set of dimensions *E*_*k*_ ⊆ *D*
Database	${\mathscr{D}}=\{{T}_{1},{T}_{2},\ldots ,{T}_{max}\}$ all transactions	${\mathcal E} =\{{E}_{1},{E}_{2},\ldots ,{E}_{max}\}$ all multidimensional edges
Frequent itemset	*C* ⊆ *T* is referred to as the frequent itemset *s*_*T*_(*C*) ≥ *s*_*min*_	*C* ⊆ *E* is referred to as the frequent dimension set *s*_*T*_(*C*) ≥ *s*_*min*_
Association rule	*A* ⇒ *B*, where *A* and *B* are disjoint sets of items; *A*: antecedent, *B*: consequent	
	*A* ⊂ *I*, *B* ⊂ *I* are sets of items, with *A*∩*B* = ∅	*A* ⊂ *D*, *B* ⊂ *D* are sets of dimensions, with *A* ∩ *B* = ∅
Support of a rule	*s*_*T*_(*A* ⇒ *B*) = *P*(*A* ∪ *B*)	
	probability that a transaction contains *A*∪*B*	probability that a multidimensional edge contains *A*∪*B*
Confidence	${c}_{T}(A\Rightarrow B)=P(B|A)=\frac{P(A\cup B)}{P(A)}=\frac{{s}_{T}(A\cup B)}{{s}_{T}(A)}$	
	probability of finding *B* under the condition that transactions also contain *A*	probability of finding *B* under the condition that multidimensional edges also contain *A*
Lift	$l=lift(A\Rightarrow B)=\frac{P(A\cup B)}{P(A)P(B)}$	
	*B* increases (lift) the likelihood of *A* if *l* < 1 negative correlation; *l* = 1 independent; *l* > 1 positive correlation exists between *A* and *B*	
Leverage	$\lambda =leverage(A\Rightarrow B)={s}_{T}(A\cup B)-{s}_{T}(A){s}_{T}(B)$ how much more often *A* and *B* occur together than expected under independence	

The resultant frequent itemsets can be used to form *A*⇒*B* association rules where *A* and *B* are disjoint subsets of *C*, as *A* ⊂ *C*, *B* ⊂ *C* and *A* ∩ *B* = ∅^43^. The confidence of the rule represents the *P*(*B*|*A*) conditional probability:

1 $${c}_{T}(A\Rightarrow B)=P(B|A)=\frac{P(A\cup B)}{P(A)}=\frac{{s}_{T}(A\cup B)}{{s}_{T}(A)}=\frac{{\rm{count}}(A\cup B)}{{\rm{count}}(A)}$$

when *A* is independent of *B*, *P*(*A* ∪ *B*) = *P*(*A*)*P*(*B*). The lift *l* is a correlation measure that is based on the ratio of these probabilities:

2 $$l=lift(A\Rightarrow B)=\frac{P(A\cup B)}{P(A)P(B)}=\frac{{s}_{T}(A\cup B)}{{s}_{T}(A){s}_{T}(B)}$$

when *l* < 1 *A* is negatively correlated with *B*, meaning that the occurrence of *A* leads to the absence of *B*. When *l* > 1, then *A* and *B* are positively correlated, meaning that the occurrence of *A* implies the occurrence of *B*^44^. Rules with high level of lift usually exhibit relatively low degree of support^45^. An alternative to lift is leverage that states how much more often *A* and *B* occur together than as independent random variables^46^.

3 $$\lambda =leverage(A\Rightarrow B)={s}_{T}(A\cup B)-{s}_{T}(A){s}_{T}(B)$$

The computational complexity of the proposed methodology is determined by the utilized frequent itemset mining algorithm. The complexity of the most widespread Apriori algorithm is ${\mathscr{O}}({M}^{2}m)$^47^, where *M* represents the number of items and *m* the number of data records, thus finding the frequent connection types has quadratic dependence on the *M* connection types and linear scalability in the $m=|{E}_{k}|$ number of connection. As *M* = 15, it can be concluded that the calculation of the proposed measures can be computed very quickly even for large networks.

In this section, an analogy between the measures of network science and frequent pattern mining was presented. In the following section, how frequent itemsets and association rule mining can be used to understand the formation of connections is demonstrated.

### Node characterization based on incoming multidimensional edges

In (organizational) network research, three levels (dyadic, actors/nodes, networks) of the analysis can be distinguished^48^. At the dyadic level the frequent occurrence of the edge dimensions can be analyzed. Analysis at the level of the actors requires information to be aggregated with regard to the types of edges to characterize the nodes. For example, to measure the degree of innovation and problem-solving abilities of the employees, the centrality of the actors in the communication network of the organization can be studied^49^. The selection of suitable dimensions plays an important role in these ratings, e.g. as information exchange is reflected in the advice network, the perception of information access is mostly determined by the advice centrality^4^. In multilayer networks, nodes can be characterized based on their activities at different layers^26^. The distribution of degrees of nodes among layers can be described by its entropy of the multiplex degree which is similar to the multiplex participation coefficient published in ref.^34^.

In the following a novel method for the characterization of nodes is introduced by calculating the frequent patterns of the incoming/outgoing multidimensional edges of ego-networks. The directed edge set ${ {\mathcal E} }_{{u}^{out}}=\{{E}_{1},{E}_{2},\ldots ,{E}_{max}\}$, ${E}_{k}=\{({u}^{out},{v}^{in},d);u,v\in V,d\in D\}$ consist of outgoing edges of a node *u* ∈ *V*; and ${ {\mathcal E} }_{{u}^{in}}=\{{E}_{1},{E}_{2},\ldots ,{E}_{max}\}$, ${E}_{l}=\{({v}^{out},{u}^{in},d);u,v\in V,d\in D\}$ represents the incoming edges of a node *u* ∈ *V*. Frequent dimensions of outgoing and incoming edges are specific to the nodes. The outgoing edges are related to the perceptions, ratings and connections to others, while the incoming patterns reflect how the actor is rated. Association rules *A* ⇒ *B* valid for ${ {\mathcal E} }_{{u}^{out}}$, ${ {\mathcal E} }_{{u}^{in}}$ or ${ {\mathcal E} }_{u}={ {\mathcal E} }_{{u}^{out}}\cup { {\mathcal E} }_{{u}^{in}}$ provide the specifications of the node.

As a node can support or weaken association rules with its incoming/outgoing multidimensional edges, the measures of the association rules can be utilized as fingerprints of the organizational network. The similarity between the nodes can be evaluated based on the incoming and outgoing patterns. Based on clustering of the nodes, similar key persons and leaders can also be identified which approach is similar to frequent pattern mining-based community detection^50^.

As modularity is based on the difference between the actual and expected number of edges^51^, the analysis of this difference can reflect attractiveness and talent in individual and organizational levels. Community detection algorithms explore densely linked groups of nodes, so these algorithms can highlight central nodes^52^, leaders of communities^53^ and hierarchical structure^54,55^.

The following section demonstrates that based on the similarities of the multidimensional incoming and outgoing connections the clusters of co-workers can be determined and use extracted knowledge can be used to characterize typical roles in the organizations.

## Results and Discussions

To demonstrate the applicability of the proposed methodology, leaders and key persons are identified based on the incoming edges and the determination of the effects with regard to the advice network based on frequent patterns containing the advice (L1) dimension are sought.

### The studied organizational networks

**Figure 2:**
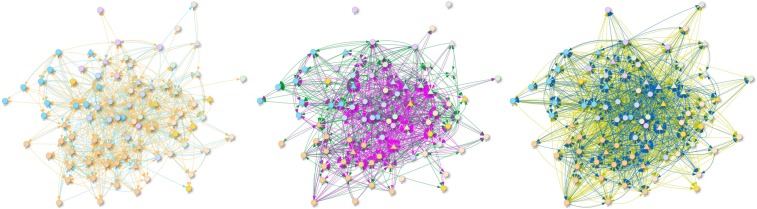
Six layers of the organizational network of Company A (left: light blue is L1, orange is L4; middle: dark green is L8, magenta is L12; right: dark yellow is L13, dark blue is L15. The nodes are colored according to the departments they belong to. The shape of nodes corresponds to the positions as triangles represent leaders and circles stand for the employees).

83 (response rate (RR): 75%), 57 (RR: 93%) and 203 (RR: 94%) employees from A: a not-for-profit arts organization, B: a multinational manufacturing company, and C: a cultural institute, respectively were studied. The complexity of Company A is illustrated in Fig. 2. The number of nodes and edges with their support is shown in Table 2. The high support of L13 in Company A indicates a friendly atmosphere. The reciprocity in the L13 layer is 43–44% for all companies, which correlates well with other studies^4^.

**Table 2:** Support values of the edge labels in the studied organizations.

	Company A	Company B	Company C
Number of nodes	83	57	203
Number of multidimensional edges	1709	925	5766
P(L1)	0.200	0.310	0.280
P(L2)	0.063	0.163	0.132
P(L3)	0.199	0.166	0.187
P(L4)	0.304	0.403	0.362
P(L5)	0.274	0.356	0.349
P(L6)	0.253	0.308	0.284
P(L7)	0.415	0.345	0.341
P(L8)	0.315	0.384	0.349
P(L9)	0.103	0.171	0.146
P(L10)	0.150	0.225	0.215
P(L11)	0.088	0.251	0.162
P(L12)	0.221	0.329	0.268
P(L13)	0.714	0.484	0.363
P(L14)	—	0.428	0.339
P(L15)	0.301	0.471	0.323

The number of two or more dimensional edges is shown in Table 3 which indicates that the majority of edges are multidimensional, only 26–33% of the edges are one-dimensional. The dimensions L4, L8 and L13 (55% of the one-dimensional in Company A), as well as the L14 and L15 tend to appear alone.

**Table 3:** Proportion of the number of dimensions in multi-edges.

	1	2	3	4	5	6	7	8	9	10	11	12	13	14	15
Company A	0.325	0.189	0.124	0.078	0.060	0.056	0.044	0.035	0.021	0.013	0.020	0.015	0.007	0.012	-
Company B	0.261	0.146	0.114	0.085	0.063	0.057	0.038	0.041	0.038	0.029	0.028	0.023	0.025	0.023	0.030
Company C	0.290	0.179	0.119	0.099	0.063	0.057	0.041	0.035	0.027	0.025	0.024	0.016	0.014	0.013	0.015

### Analysis of the extracted association rules

Finding meaningful association rules is one of the biggest challenges in data mining. Filtering the rules based on confidence and support is an obvious approach, but in some cases, the grouping of the rules based on variables is necessary^56^, e.g., the setting of a high-threshold support would exclude rare dimensions from the rules (like L2 and L9).

A positive correlation is indicated between the antecedent and consequent sets of all rules with a lift greater than one. Only two rules exist in Company A that possess negative leverages. The L8 ⇒ L13 rule can be found on 370 edges that is less than 380 edges expected under independent conditions, which indicates on average that it is hard to get along well with people who possess a high degree of professional knowledge.

**Figure 3:**
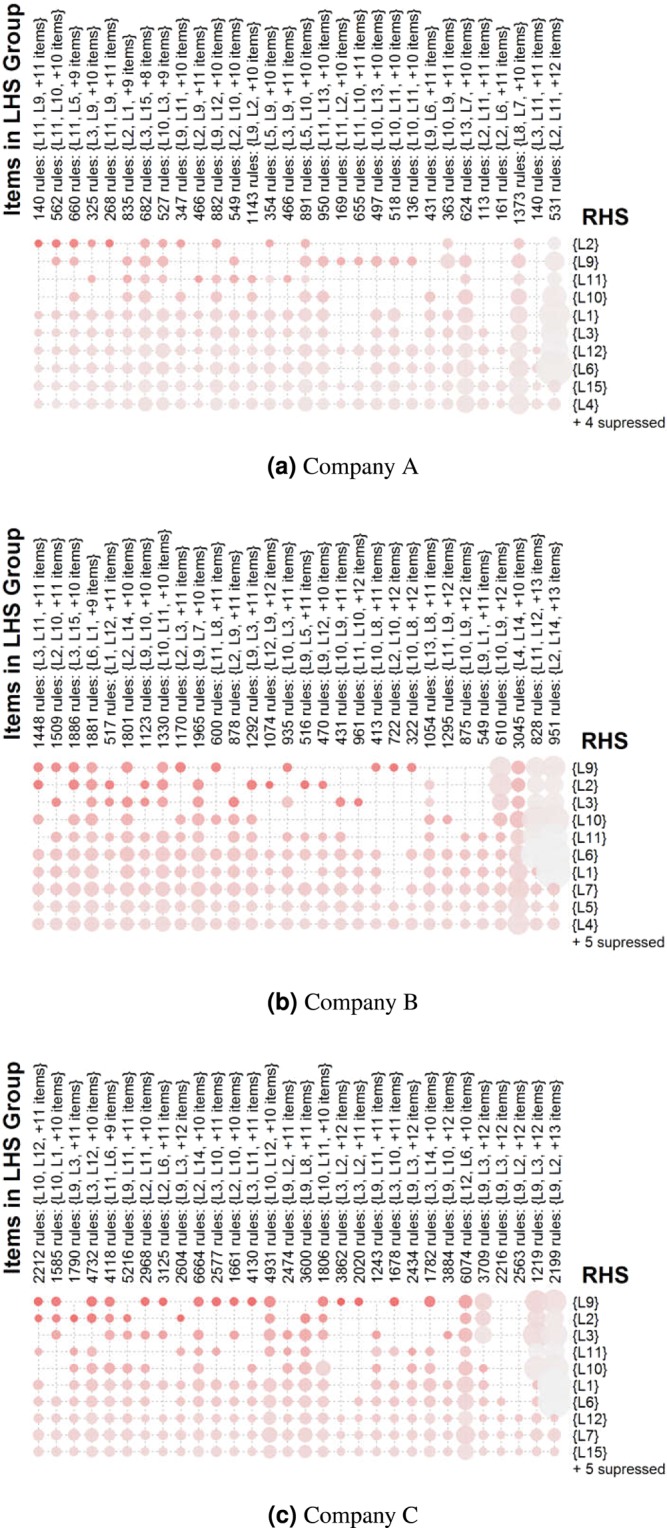
Summary of rules (size is proportional to support, color is proportional to lift).

The extracted rules as grouped matrices^45^ are summarized in Fig. 3, where the antecedents that are statistically dependent on the same consequents are grouped and shown in columns with their two most frequent dimensions written on the axes. Consequents are arranged in the rows. The bubbles are colored according to the median lift of the rules in the groups, while the sizes of the bubbles represent the medians of the supports. The resultant plots highlight that important consequents are very similar in all companies, namely L2, L9, L11, L3, L10 and L1 which refer to leadership, motivation, managerial capability, giving feedback, solving complex tasks and giving advice respectively.

**Figure 4:**
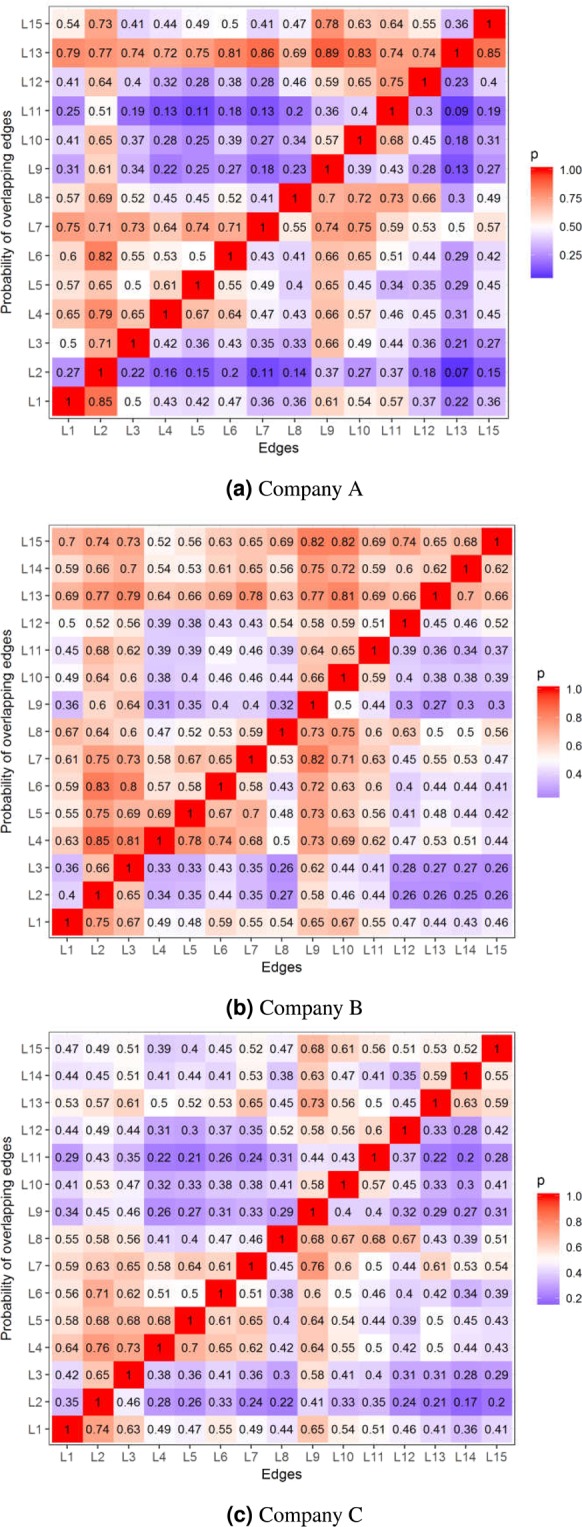
The probability of dimensions in rows given the dimensions in the columns in the case of all three companies.

The confidence values of the rules can serve as layer-overlap measures. In Fig. 4 the columns are antecedents and the rows are consequents of rules *Column* ⇒ *Row*, furthermore, the values of the matrix show the confidences of the rules. As expected, layers L2 and L9 exhibit a strong correlation between almost all other layers, while it seems that the precedences of the edges L9, L10 and L11 increase the probabilities of connection types L7, L8 and L12.

### Characterization of the leaders

The appearance of dimension L2 in a multidimensional edge shows who is considered to be a leader in an organization because he/she provides instruction in a workflow. The confidences of the leader-related rules are shown in the second columns of the matrices in Fig. 4. The *c*(*L*2 ⇒ *L*9) = *P*(*L*9|*L*2) confidence of the *L*2 ⇒ *L*9 rule is a good measure of how a co-worker perceives the motivation of his/her leader.

**Figure 5:**
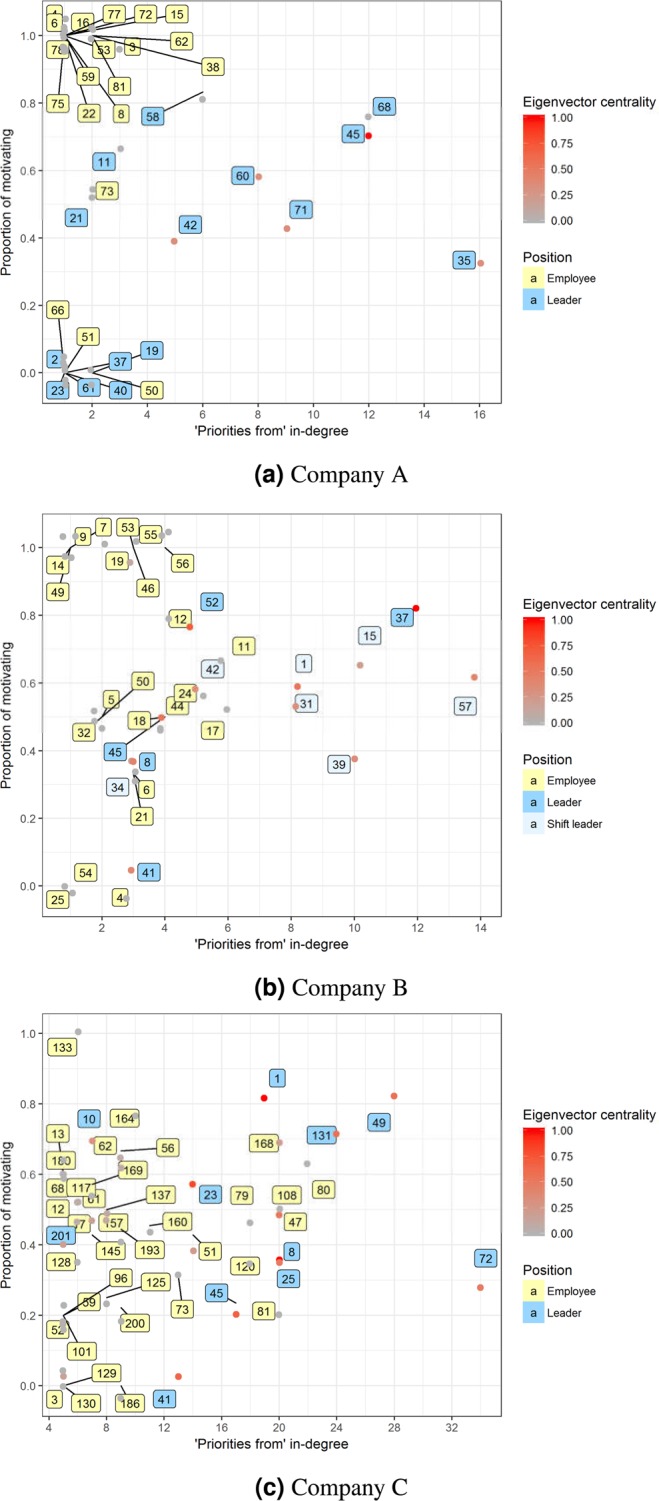
Motivating leaders. For the sake of interpretability of the figures, a small amount of random variation is added to the location of each point to avoid overlapping and persons with more than four in-degree are plotted at Company C.

The two-dimensional evaluation of actors is represented in Fig. 5. The x-axis is the in-degree on the “priorities from” (L2) layer, and the y-axis shows the conditional probability of the presence of “motivates me” (L9) dimension along the same L2 edge. The in-degree centrality does not correlate with the motivating capability (Pearson’s *ρ* between the in-degrees and the motivating capability of the nodes is 0.38 at Company A; −0.09 at Company B; and 0.21 at Company C), so the two dimensions provide additional information about actors. However, high and low social capital correlate with the in-degree centrality which reflects the eigenvector centrality captures the importance of the actors^57^. Eigenvector centralities of actors on the L2 layer are also well correlated with in-degrees (Pearson’s *ρ* between the in-degree and the eigenvector centrality of the nodes is 0.71 at Company A; 0.68 at Company B; and 0.67 at Company C). The differences in the eigenvector centrality among actors with the same in-degree can be studied in Fig. 5. The leader numbered as ‘45’ in Company A has much higher eigenvector centrality than leader numbered as ‘68’, but they have the same motivating capability that indicates that leader ‘45’ motivates more important people than leader ‘68’ which increases his/her overall importance.

The fact that there is no correlation between the numbers of motivation type connections and eigenvector centrality (Company A: 0.11; Company B: −0.06; Company C:0.17) shows that the capability of motivating may a personal trait. The plots can be utilized to evaluate the performance of the leaders and support decisions related to organizational development.

### Clustering-based identification of the key persons

**Figure 6:**
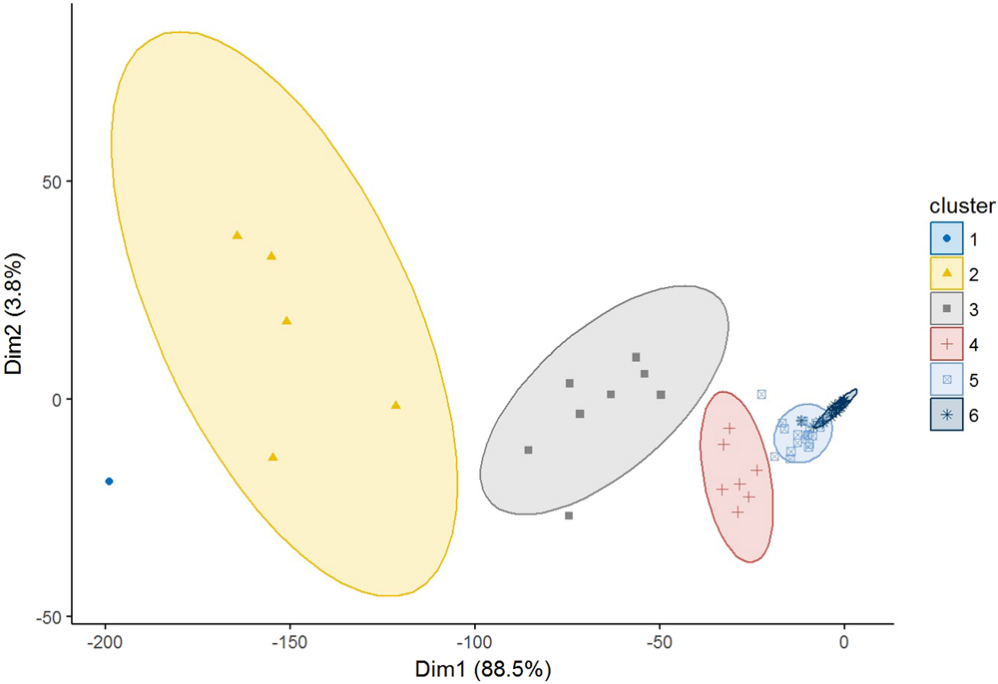
Clusters of key persons in Company A visualized by principal component analysis. The numbers at the axes labels show the percentage of the variance represented by the principal component.

Finding influential employees in organizations should differ from the analysis of formal organizational charts. Research questions like “who is considered to be a key person?” require detailed analysis. A clustering-based algorithm to answer such questions was developed. Similarly evaluated people can be clustered based on how similarly their incoming edges support the association rules. The Partitioning Around Medoids (PAM) algorithm^58^ was applied to identify the clusters (see Fig. 6) as it lends itself to clustering based on the specified distance matrix^59^, it has the robustness to noise^60^ and performs better for large datasets than the also popular k-means algorithm^61^.

Clusters 1 and 2 include the top managers of Company A. People in Clusters 3 and 4 are evaluated as key persons to the same extent. Members of Cluster 3 advise more co-workers, while members of Cluster 4 are evaluated as possessing greater professional knowledge. 38% of Cluster 3 are middle managers. There is no leader in Cluster 4, which suggests that members select advisors based on their status^13^.

### Effects of the advice network

According to the literature, at least two kinds of processes drive how sources of advice are selected: namely status recognition and homophily^13^. The extraction of valuable information concerning these effects based on the analysis of the Lift(*L*1 ⇒ *B*) values was attempted.

Table 4 shows that the edge types of leadership (L2 and L3), motivating behavior (L9), information resources (L10) and cognitive ability (L6) increase the likelihood of leadership (L1). Although there are some specificities in term of the networks of different companies, e.g. lift(*L*1 ⇒ *L*2) is much greater in the case of Company A, but its trends are very similar. The high confidence values in the L1 columns of Fig. 4 indicate that this connection type has positive effects on contact type, trusted relationships and the judgment of professional knowledge.

**Table 4:** Lift(*L*1⇒*B*) values at the studied companies.

	Company A	Company B	Company C
L2	4.25	2.43	2.63
L3	2.51	2.16	2.25
L4	2.14	1.56	1.76
L5	2.08	1.55	1.67
L6	2.36	1.91	1.96
L7	1.82	1.78	1.74
L8	1.80	1.73	1.57
L9	3.04	2.10	2.34
L10	2.72	2.17	1.93
L11	2.83	1.78	1.81
L12	1.84	1.52	1.62
L13	1.11	1.42	1.47
L14	—	1.38	1.29
L15	1.80	1.48	1.46

Dimensions that predict the occurrence of L1 are described by the *A* ⇒ *L*1 family of rules. The confidence(*A* ⇒ *L*1) of these rules represents the probability of the occurrence of L1 given the existence of the *A* dimensions. As is shown in Table 5, professional knowledge (L8) leads to a far more significant increase in the probability of get advice dimension (L1) than communication (L4) in the case of the existence of a leader (L2), working relationships (L5) and best working relationship (L7), as well as information sources (L6). However, L4 significantly increases the probability of the advice-type connection when motivation (L9), the capability of solving complex tasks (L10), the ability to manage colleagues (L11), and key person (L12) exist. In other words, the confidences of {L2 or L5 or L6 or L7} ∪ L8 ⇒ L1 are greater than the confidences of {L9 or L10 or L11 or L12} ∪ L8 ⇒ L1, and the confidences of {L2 or L5 or L6 or L7} ∪ L4 ⇒ L1 are less than the confidences of {L9 or L10 or L11 or L12} ∪ L4 ⇒ L1.

**Table 5:** Confidences of the rule *A*⇒*L*1 of the companies.

	Company A				Company B				Company C			
Antecedents (A)		∪ L4	∪ L8	∪ L8 ∪ L9		∪ L4	∪ L8	∪ L8 ∪ L9		∪ L4	∪ L8	∪ L8 ∪ L9
L2	0.850	0.882	0.919	0.943	0.755	0.797	0.907	0.915	0.735	0.816	0.873	0.950
L3	0.502	0.683	0.655	0.761	0.668	0.733	0.847	0.901	0.630	0.736	0.813	0.883
L4	0.428		0.637	0.806	0.485		0.761	0.839	0.492		0.747	0.868
L5	0.415	0.503	0.582	0.752	0.480	0.558	0.703	0.784	0.466	0.567	0.745	0.869
L6	0.472	0.594	0.627	0.806	0.592	0.674	0.807	0.840	0.550	0.660	0.769	0.864
L7	0.363	0.549	0.554	0.780	0.551	0.649	0.734	0.818	0.488	0.617	0.713	0.850
L8	0.360	0.637			0.538	0.761			0.440	0.747		
L9	0.607	0.752	0.693		0.651	0.730	0.756		0.654	0.812	0.772	
L10	0.544	0.746	0.616	0.764	0.673	0.797	0.722	0.827	0.540	0.763	0.649	0.832
L11	0.566	0.913	0.651	0.854	0.551	0.717	0.685	0.804	0.505	0.791	0.599	0.822
L12	0.368	0.621	0.473	0.722	0.470	0.715	0.637	0.766	0.455	0.744	0.567	0.819
L13	0.221	0.486	0.462	0.734	0.441	0.635	0.651	0.795	0.411	0.626	0.635	0.812
L14					0.426	0.625	0.653	0.806	0.360	0.628	0.622	0.834
L15	0.361	0.571	0.549	0.754	0.458	0.716	0.648	0.805	0.409	0.672	0.605	0.811

Most leaders (L2) give advice, especially in Company A. The probabilities are increased as the dimensions of the rules increase. Almost the same trends are shown in Tables 4 and 5 where the types of connections that are related to the advice network are presented. This result correlates well with the findings of ref.^13^ which show that advice is more likely to be sought from colleagues of higher statuses.

## Conclusions

Organizational networks have been considered to be multilayer networks since the early 1990s, but so far no feasible method of handling their multidimensionality has been found. It has been demonstrated that frequent pattern mining can be applied to reveal statistically significant correlations between the layers and that the method is applicable regarding edge, actor and organizational level analyses. Frequently occurring outgoing edges have been shown to be related to perceptions and ratings, while incoming patterns reflect how the actor is rated. It was also highlighted that measures of the association rules could be used to define the fingerprints of organizational networks. The applicability of the methodology was demonstrated by the characterization of leaders and key persons in three organizations. In the future, the utilization of an extracted rule-base for the design of personal development programs, and the determination of a property-preserving multidimensional edge reordering algorithm to support goal-oriented organizational development is desired. The method can be applied to other multilayer networks where layers can represent dimensions and appropriate to make rankings.

## Data Availability

Original data is not available for public use.
